# A Meta-Analysis of the Relationship Between Emotional Intelligence and Academic Performance in Secondary Education: A Multi-Stream Comparison

**DOI:** 10.3389/fpsyg.2020.01517

**Published:** 2020-07-21

**Authors:** Nicolás Sánchez-Álvarez, María Pilar Berrios Martos, Natalio Extremera

**Affiliations:** ^1^Department of Basic Psychology, Faculty of Psychology, University of Málaga, Málaga, Spain; ^2^Department of Social Psychology, Faculty of Psychology, University of Jaén, Jaén, Spain; ^3^Department of Social Psychology, Faculty of Psychology, University of Málaga, Málaga, Spain

**Keywords:** emotional intelligence, academic performance, secondary education, meta-analysis, instruments

## Abstract

This study was a quantitative meta-analysis of empirical research on the relationship between emotional intelligence (EI) and academic performance (AP) that included the three main theoretical models of EI. We conducted a computerized literature search in the main electronic databases. Forty-four of an initial 3,210 articles met the inclusion criteria. With 49 effect sizes and a cumulative sample size of 19,861 participants, we found significant heterogeneity indices indicating a variety of results. In general, the results of this study indicated a significant effect of EI on AP ($\bar{Z}$ = 0.26). Average association between EI and AP was higher in studies measured EI as ability ($\bar{Z}$ = 0.31), than studies measured EI as self-report ($\bar{Z}$ = 0.24), and self-report mixed EI ($\bar{Z}$ = 0.26). In the educational field, this meta-analysis provides information on the specific role of EI as a function of used measures. Some practical implications are discussed.

## Introduction

In the educational field, academic performance (AP) is the construct that has been studied most. Teaching, learning, and all the cognitive factors related to AP have been widely examined (Pellitteri and Smith, 2007). Recently, one of the most analyzed research lines concerns the influence of personality factors and personal skills on achievement of AP (Poropat, 2009; MacCann et al., 2019). In the last 20 years, a large portion of research has been guided by a recent theoretical focus on emotional abilities, specifically emotional intelligence (EI), which has been viewed as a key component of the factors that influence well-being as well as adaptive processes in specific contexts (Zeidner et al., 2012). Several reviews showed the relevance of EI as a personal resource associated with health outcomes (Martins et al., 2010), well-being (Sánchez-Álvarez et al., 2016), and even task performance (Miao et al., 2017). Likewise, literature reviews focused on analyzing the role of EI in AP have been published (Perera and DiGiacomo, 2013; MacCann et al., 2019). These studies showed significant effects of EI in predicting AP after controlling the effects of intelligence and personality traits. In addition, EI has emerged as a strong predictor in secondary education.

### Academic Performance

Academic success or performance by students in educational centers is a key goal in the development of all educational programs. AP has been commonly measured through continuous exams or evaluations, with a general consensus about the most important aspects to evaluate, such as skills, and declarative and procedural knowledge (Ward et al., 1996). Although there is no common agreement for the evaluation of AP, measures of cognitive skills or declarative knowledge are the main factors evaluated (Perera and DiGiacomo, 2013), and the most commonly used indicators to measure AP are usually: Grade Performance Academic (GPA), Achievement Test (AT), Grade Average (GA), Academic Achievement (AA), Standard Assessment Test (SAT), and Teacher Ratings Academic (TRA) (Perera and DiGiacomo, 2013).

Recent empirical research in education regarding predictors of AP has focussed on intelligence, IQ, or personal cognitive abilities. This research movement has accumulated an extensive research literature on the measurement of cognitive intelligence (Ritchie and Tucker-Drob, 2018). Moreover, there are other personal skills that differ from traditional cognitive intelligence that could affect academic success (Furnham et al., 2009). Currently, there are several lines of research that analyse individual non-cognitive factors that increase the prediction of AP, which requires broader educational models that integrate personal and contextual factors (Gutman and Schoon, 2013). Other non-cognitive skills include attitude, motivation, personality traits, self-regulation, resilience, and social and emotional skills, which are beyond the academic skills that determine successful performance (Bowles and Gintis, 2007). Likewise, personal factors such as motivation and emotional self-regulation in the classroom are associated with school performance, that is, students who are more motivated and have greater skill to manage emotions to obtain higher academic qualifications (Pintrich and de Groot, 1990). Currently, an increasing number of studies have examined the role of emotional skills such as EI in AP.

### Emotional Intelligence

Since the EI concept was first introduced in the scientific literature by Salovey and Mayer (1990), different EI models have been developed. Based on the measurement methods used, the different theoretical conceptions of EI can be grouped into three main streams: (stream 1) Mayer and Salovey (1997) four branch ability model of EI, which defines ability EI as having four components, including the capacity to perceive, value, and express emotions accurately; the ability to access and generate feelings that facilitate thinking; the ability to understand emotions and emotional awareness; and the ability to regulate emotions and promote emotional and intellectual growth; (stream 2) cognitive emotional abilities three-branch self-perception model of Salovey and Mayer (1990), self-report EI proposes the existence of a continuous reflexive process associated with one's mood; (stream 3) cognitive emotional competences and other non-cognitive features like personal skills, motivation, and social aspects is conceived how EI mixed model (Goleman, 1995; Mayer and Salovey, 1997; Petrides et al., 2004a; Bar-On, 2006).

The ability EI stream (stream 1), also defined as EI-performance, is the conception of EI that seems to have the most similarity to AP, because EI is measured by exercises and problems to assess emotional ability, just as exams are used to measure AP in schools. On the other hand, because ability EI is assessed in a similar way to AP, students with higher levels of EI-performance could better manage stress related to exams, resulting in better AP (Brackett and Salovey, 2006). At the same time, students with inadequate or poor emotional skills will have school maladjustment, interpersonal problems that affect their anxiety (Rivers et al., 2012), and/or a lack of social support from their peers that affects their AP (Mestre et al., 2006). The instruments developed to assess ability EI, the Mayer, Salovey, and Caruso Emotional Intelligence Test (MSCEIT) (Mayer et al., 2002) and the Multifactor Emotional Intelligence Scale (MEIS) (Mayer et al., 1999), have objective criteria for correct and wrong answers.

The self-report EI stream (stream 2), based on self-perception of one's emotional skills, assesses a person's subjective emotional abilities. This means that each individual indicates their level of EI according to their previous experiences and their level of self-esteem, including the mood in which they find themselves when completing the EI self-report scale (Davies et al., 1998). This type of measure is usually related to well-established personality factors such as neuroticism, extraversion, agreeableness, openness, and psychoticism, and this connection can yield false correlations with performance and academic achievement (Gannon and Ranzijn, 2005). Representative self-report EI instruments include the Wong and Law Emotional Intelligence Scale (WLEIS) (Wong and Law, 2002), Trait Meta-Mood Scale (TMMS) (Salovey and Mayer, 1990), Schutte Emotional Intelligence Scale (SEIS) (Schutte et al., 1998; Saklofske and Zeidner, 2006), and Swinburne University Emotional Intelligence Test (SUEIT) (Palmer and Stough, 2001).

In the mixed EI stream (stream 3), the integration of different personal and social skills leads to overlapping effects with other factors that may influence AP. When evaluating personality variables, cognitive skills, and social-emotional traits together, one obtains a profile that may be more associated with the different skills that are implemented in an academic context. Therefore, students with better social-emotional traits, with high cognitive abilities (Shen and Comrey, 1997), and adaptive personality trait variables achieve better test scores (Pulford and Sohal, 2006; Poropat, 2009). Therefore, students with better adaptation to the school context will obtain better scores in AP than students with profiles less oriented toward academic adaptation. Representative measures of mixed EI include the Emotional Quotient Inventory (EQi) (Bar-On, 1997), Trait Emotional Intelligence Questionnaire (TEIQ) (Petrides, 2009), and Emotion Identification Skills (EIS) (Ciarrochi et al., 2008).

Each of the three main streams has contributed to research linking EI and AP, with heterogenous results, despite being evaluated with instruments developed under the same theoretical conceptions of EI. It is not surprising that EI is conceived from several theoretical approaches. A possible cause of the lack of consensus on the results may be the multitude of instruments to evaluate EI from the different theoretical approaches.

### Theoretical Linkages Between Emotional Intelligence and Academic Performance

The EI literature has shown that individuals with a higher capacity to process information typically perform better on cognitive tasks (Saklofske et al., 2012). Interpersonal and intrapersonal skills are of great importance in secondary education, since it is a period that involves many social, contextual, and personal changes and stresses. During adolescence, the peer group is of great relevance to adolescents' emotional development and identity formation (Duncan et al., 2006; Eccles and Roeser, 2009), with immediate contexts such as the school environment being one of the most relevant (Monreal and Guitart, 2012). In this sense, the events and early experiences lived in the different contexts, the reactions and responses of adolescents to the different situations of risk and stress throughout their development, as well as the existence of resource vulnerability protection, are relevant and important to understanding individual differences between young people (Monreal and Guitart, 2012). Greater emotional regulation and a better process of adaptability are useful to cope with academic stress and achieve academic success (Saklofske et al., 2012). Interestingly, emotional perceptive people appear to be more strongly impacted by stress than their less perceptive counterparts, expressing higher levels of psychological distress (Ciarrochi et al., 2002). It is hypothesized that low perceptive people might ignore thoughts of daily hassles and therefore might be more likely to be confused about the experienced negative feelings showing less coherence between their levels of perceived stress and psychological maladjustment. Thus, people with high EI are more resilient, adapting more easily to changes, reacting better under stress conditions, and coping with difficulties in the form of challenges (Schneider et al., 2013). Finally, students with a better management of their emotions are happier and have better social relationships (Eryilmaz, 2011). In turn, having better interpersonal management is generally associated with higher social networks, as well as better friendships quality (Brackett et al., 2005). Similarly, having a greater social network in a classroom might stimulate an adequate social environment for better cooperative work, better group learning, greater support from classmates (Hogan et al., 2010), and better relationships with teachers (Di Fabio and Kenny, 2015). Together, both the academic climate involving classmates and professors, as well as a better predisposition of learning-oriented abilities might be associated with a greater AP (Brackett et al., 2011; Johnson, 2016). In summary, there are several plausible theoretical mechanisms that might explain the relationship between EI as a set of skills and optimal academic functioning in secondary education.

### Current Meta-Analysis

Previous work has excluded studies conducted with instruments developed under other theoretical approaches of EI (Perera and DiGiacomo, 2013), or has contemplated the role of EI in AP in a more global way and by levels (MacCann et al., 2019), making it difficult to compare the results between different instruments. The present study examined the association between EI and AP, considering instruments developed from all the theoretical approaches to EI in studies conducted in secondary school students, as an educational level of greater relevance according to previous literature (Perera and DiGiacomo, 2013; MacCann et al., 2019). Our meta-analysis aimed to examine previous review studies, comparing the results by the main streams and EI instruments used in secondary education including native English and Spanish speakers. The current meta-analysis study was carried out to (1) asses the associations of AP and EI, hypothesizing that there will be a significant correlation between EI; (2) show the associations of different instruments used to assess EI based on three main streams and levels of AP; in line with previous studies, it was hypothesized that EI ability instruments would have a greater association with AP.

## Methods

### Literature Search

We searched relevants studies of EI y AP on electronic database: PsychoINFO, MEDLINE, SCOPUS, PubMed, ISI Web of Science, Google Scholar, and ProQuest Dissertations and Theses. The search term (emotional intelligence) AND (academic performance OR academic achievement OR grades performance OR academic OR education OR school) AND (secondary level). We also reviewed specialized database journals of relevant papers. This review was conducted from June 2017 to January 2020.

### Inclusion Criteria

Studies eligible were scanned titles and abstracts, and included in the review all those that referred to the above terms. To be included in the review, papers had to meet the following inclusion criteria for eligibility of studies (Lipsey and Wilson, 2000): (1) empirical study that provides data on the association between EI and AP; (2) minimum sample size at least 20 participants; (3) studies had to have been performed between 1999 and 2020 (January); published article and unpublished doctoral thesis without published and conference paper, (4) studies written in Spanish and English.

### Coding

Following a Lipsey and Wilson (2000) : (a) country, (b) publication type, (c) design features, (d) measure used to asses EI, (e) AP index, (f) study sample size, (g) size of the association between key variables, (h) level of significance. Finally, extrinsic characteristics coded were results reporting the year and publication source (see Table A1).

### Statistical Analysis

All data were conducted in R (Team, 2012), using the “stats” and “metaphor” packages (Viechtbauer, 2010). For the meta-analysis the technique by DerSimonian and Laird (1986) was used. The *Q*-value indicated heterogeneity among studies (*p* < 0.10), thus applied random effect models was used in the meta-analysis. Additionally, we quantified the effect of heterogeneity using *I*^2^ (Higgins and Thompson, 2002). The *I*^2^ value indicate proportion of inconsistency due to heterogeneity rather than chance. The effect size index was converted by Fisher r – Z following the procedures recommended by Hedges and Olkin (1985). The categorical model between-class results were obtained through a goodness-of-fit statistic *Q*_*b*_, and the within-class goodness-of-fit statistic *Q*_*w*_. The statistic *Q*_*wj*_ within-category heterogeneity is under the null hypothesis of within-category homogeneity.

### Publication Bias

Publication bias was evaluated by rank correlation with Kendall's tau method, in which a significant correlation indicates publication bias, and Egger's regression test asymmetry, in which significant asymmetry indicates publication bias (Fernández-Castilla et al., 2019). The Egger regression test should not differ significantly (*z* = −1.189, *p* = 0.234), and the rank correlation yielded non-significant results (*T* = 0.03, *p* = 0.243). Non-significant results showed symmetry and absence of publication bias. Regression tests and the funnel plot indicated a non-significant asymmetry, so the results showed no evidence of publication bias between EI and AP.

## Results

### Selected Studies

**Figure 1:**
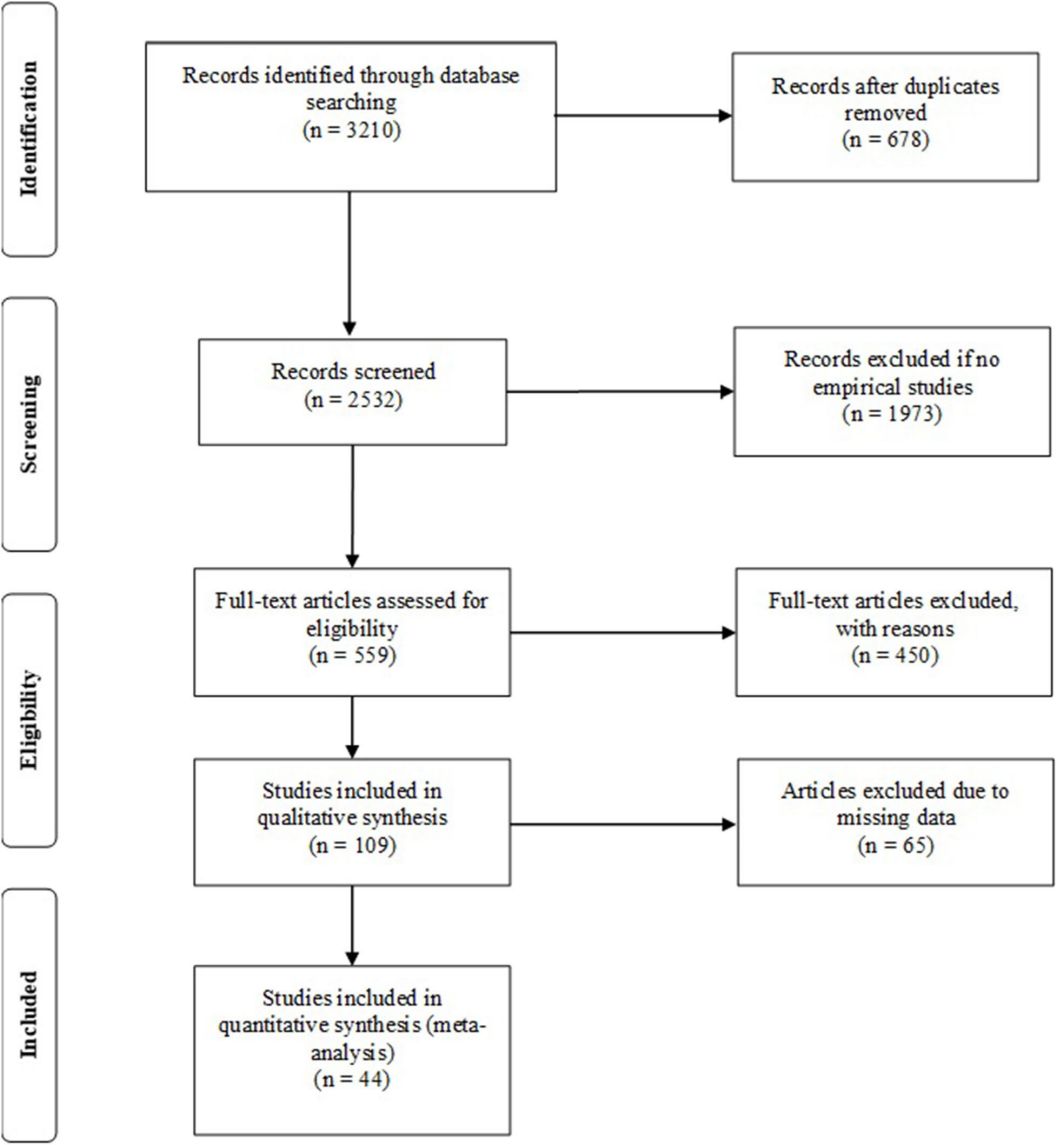
PRISMA flowchart for the identification, screening, and inclusion of publications in the meta-analyses.

The sample consisted of 3,210 studies, 678 were duplicate studies. Eventually, 1,973 did not correspond to association between EI and AP. They associated lack of personal distress and absence of mental disorder to higher levels of well-being. The full text of the remaining 559 articles were reviewed, obtaining 44 items that were selected and evaluated more deeply (see Figure 1).

### Study Characteristics

The articles included in the meta-analysis showed a closed association between target variables. The overall sample consisted of 19,714 participants, and the mean age was of 15.82 years. Several studies included some scales for assessing EI, obtaining 49 effect sizes. The studies included were conducted in 16 countries, with the largest number conducted in the US (14 studies).

### Association of EI and AP

The main results of this study indicated that the association between EI and AP had a significant low to moderate cumulative effect ($\bar{Z}$ = 0.26; CI from 0.14 to 0.38). A DerSimonian test and Laird's random effect showed statistical evidence of heterogeneity (*Q* = 1,206.16, *p* < 0,001), indicating a greater variance of effect sizes between studies than anticipated by chance. In addition, the *I*^2^ estimated of 96% suggests a high proportion of variation between samples.

### Main EI Streams

The categorical model test that examined the subgroup model results intra-group showed statistical evidence of heterogeneity (*Q*_*b*_= 0.39, *p* = 0.540). The *Q*_*w*_ statistics revealed that the model was misspecified (*Q*_*w*_= 1,205.77, *p* < 0.001). Therefore, significant differences were found between the effect sizes, indicating heterogeneity within each category (see Table 1). The ability stream showed lower levels of heterogeneity (*Q*_*wj*_= 24.16, *p* < 0.012), with smaller variation between scores (*I*^2^ = 54%) obtained between the different studies that used ability stream instruments. When examining the effect size results by grouping the EI instruments by main streams, we found larger effect sizes for those studies that used instruments based on the ability EI stream ($\bar{Z}$ = 0.31). At the same time, the degree of inconsistency between studies that used instruments based on the ability EI stream was lower (*I*^2^ = 54%) than in the other groups of studies (self-report EI stream *I*^2^ = 99%; mixed EI stream *I*^2^ = 92%).

**Table 1:** Olkin and Pratt weighted average ($\bar{Z}$), effect size number (K), homogeneity test (*Q*_*wj*_), and the degree of inconsistency (*I*^2^) between EI main stream.

**EI stream**	***N***	***$\bar{Z}$***	***K***	***Q_***wj***_***	***P***	***I*^**2**^ %**
Ability (stream 1)	2,644	0.31	12	24.16	0.012	54
Self-report (stream 2)	9,529	0.26	7	869.24	0.001	99
Mixed (stream 3)	7,628	0.24	25	312.37	0.001	92

*Q_w_ = 1205.77, p < 0.001*.

*Q_b_ = 0.39, p < 0.100*.

### Type of EI Measure

As shown in Table 2, the different instruments used to assess EI had differing levels of association with AP. Moreover, there was much variability in the scores obtained in studies using the same EI instrument. Only the MSCEIT (*Q*_*wj*_= 3.05, *p* = 0.880), SUEIT (*Q*_*wj*_= 0.63, *p* = 0.426), and Situational Test of Emotion Management for Youths (STEM-Y) (*Q*_*wj*_= 0.51, *p* = 0.476) measures did not show significant levels of heterogeneity between the effect sizes of the different studies. On the other hand, the largest effect sizes were observed in studies that used the Behavior Emotional Quotient Inventory (EQBI) ($\bar{Z}$ = 0.94, *K* = 1), followed by the studies carried out with the MEIS ($\bar{Z}$ = 0.50, *K* = 1), EIS ($\bar{Z}$ = 0.40, *K* = 5), and MSCEIT ($\bar{Z}$ = 0.35, *K* = 8) instruments. At the same time, the lowest degree of inconsistency between studies that used the same instruments was found for the SUEIT (*I*^2^ = 58%, *K* = 2), followed by the MSCEIT (*I*^2^ = 78%, *K* = 8), and Emotional Quotient Inventory (EQ-i) (*I*^2^ = 82%, *K* = 15), with the EQ-i being the most widely used instrument.

**Table 2:** Olkin and Pratt weighted average ($\bar{Z}$), effect size number (K), homogeneity test (*Q*_*wj*_), and the degree of inconsistency (*I*^2^) between EI measure.

**EI measure**	***N***	***$\bar{Z}$***	***K***	***Q_***wj***_***	***P***	***I*^**2**^ %**
MSCEIT	138	0.35	8	3.05	0.880	78
EQ-i	3,017	0.21	15	80.58	0.001	82
TEIQue	2,675	0.18	8	92.30	0.001	92
SUEIT	452	0.22	2	0.63	0.426	58
AMEIS	205	0.14	1	–	–	–
EIS	1,936	0.40	5	62.89	0.001	93
EQBI	1,563	0.94	1	–	–	–
MEIS	39	0.50	1	–	–	–
ESCQ	380	0.17	1	–	–	–
SSREI	127	0.15	1	–	–	–
TMMS	5,268	0.17	2	12.17	0.001	91
STEM-Y	525	0.26	2	0.51	0.476	97
PSM	486	0.14	1	–	–	–
EM	1,799	0.02	1	–	–	–

*Q_w_ = 252.13, p < 0.001*.

*Q_b_ = 954.04, p < 0.001*.

### Type of AP Measure

Subgroup analysis was conducted to examine the variability in the scores obtained in studies using the same AP instrument (see Table 3). The highest degree of variability in the scores between studies using the same instruments was found for the GPA (*Q*_*wj*_ = 246.68, *p* < 0.001), AA (*Q*_*wj*_ = 16.35, *p* = 0.003), and GCSE (*Q*_*wj*_ = 35.07, *p* < 0.001). Furthermore, the largest effect sizes were observed in studies using the WAEC ($\bar{Z}$ = 0.74, *K* = 1), followed by the studies using the VSLECRA ($\bar{Z}$ = 0.38, *K* = 1), and GPA ($\bar{Z}$ = 0.28, *K* = 30) instruments. Simultaneously, the lowest degree of inconsistency between studies using the same instruments was found for the TRA (*I*^2^ = 47%, *K* = 2), followed by the AA (*I*^2^ = 76%, *K* = 5) and GPA (*I*^2^ = 88%, *K* = 30), with the GPA being the most widely used instrument.

**Table 3:** Olkin and Pratt weighted average ($\bar{Z}$), effect size number (K), homogeneity test (*Q*_*wj*_), and the degree of inconsistency (*I*^2^) between AP measure.

**AP measure**	***N***	***$\bar{Z}$***	***K***	***Q_***wj***_***	***P***	***I*^**2**^ %**
GPA	11,623	0.28	30,00	246.68	0.001	88
ACS	205	0.14	1,00	–	–	–
AA	1,337	0.24	5,00	16.35	0.003	76
ABE	161	0.20	1,00	–	–	–
AT	165	0.12	1,00	–	–	–
MA	169	−0.24	1,00	–	–	–
GA	2,168	−0.01	2,00	0.49	0.485	99
TRA	254	0.23	2,00	1.90	0.168	47
VSLECRA	142	0.38	1,00	–	–	–
WAEC	1,563	0.74	1,00	–	–	–
SAT	139	0.07	1,00	–	–	–
GCSE	1,935	0.14	3,00	35.07	0.001	94

*Q_w_ = 300.48, p < 0.001*.

*Q_b_ = 955.71, p < 0.001*.

## Discussion

The current study was designed to examine the relationship between EI and AP through meta-analyses comparing diverse main EI streams and instruments used in secondary education. Filling the gaps in previous meta-analytic research, our study provides new data, and expands past findings. After a literature review, 44 studies with 49 independent effect sizes based on 19,714 secondary school students were included in cumulative quantitative research on the link between EI and AP. Publication bias analysis showed that these findings are robust and reliable.

Regarding hypothesis 1, we found a moderate significant cumulative effect between EI and AP, including measures of the three main EI streams, and diverse indicators of AP. These findings support previous research (Perera and DiGiacomo, 2013; MacCann et al., 2019) suggesting that EI levels are moderately associated with academic success, which suggests that knowledge of one's own and others' feelings, as well as the ability to solve adaptive problems, provides an essential basis for academic learning (Zeidner and Matthews, 2016). Additionally, these results show that EI is a personal resource with an important influence in the academic field, as a process of adaptation to the environment (Zeidner et al., 2012). EI has a dual role; on the one hand, it has intrapersonal affective influences on aspects related to AP, such as motivation and self-regulation. On the other hand, interpersonal skills increase social networks in the academic environment, improving teamwork, which is so important in secondary education level. Teaching staff, through workshops can develop emotional skills to help improve mental health and interpersonal aspects, which is supported by previous literature. Current programs aim to reduce aggressive behavior and substance use; future programs should also target school performance. To deepen these interactions between emotional skills and relevant factors in AP, it would be interesting for future meta-analytical studies to focus on revealing and quantifying each of these links, especially those that are relevant at the secondary level, as it is a period full of changes, is very sensitive to risks, and involves searching for immediate well-being.

With respect to hypothesis 2, we found differences in the levels of association of EI and AP as a function of the EI measures category. The results showed non-significant differences, with ability EI measures (Mayer and Salovey, 1997) showing a greater association with AP, followed by self-report EI (Salovey and Mayer, 1990), and finally the mixed EI stream (Bar-On, 2006). This higher index of association between EI measured with ability instruments and AP may be due to similarities with the tests used to obtain AP, as both of them use performance-based tests. In this sense it is possible this collinearity effect occurred because students who have good abilities to respond to performance tests will obtain high scores in both EI tests and tests that evaluate AP (Ogundokun and Adeyemo, 2010). At the same time, and contrary to other meta-analytical studies on EI (Martins et al., 2010; Sánchez-Álvarez et al., 2016), the most commonly used instruments in academic contexts are instruments developed from the mixed EI approach. Future studies should analyse in detail these effects of overlap and collinearity with personality and other aspects to obtain non-biased findings. Previous review studies (Perera and DiGiacomo, 2013; MacCann et al., 2019) did not assess the impact of different measures of EI on the association with AP, so these findings provide relevant information for future studies. The results showed great heterogeneity within each instrument category, presenting large differences between different studies that used the same instrument to measure EI (Sánchez-Álvarez et al., 2016). This variability could be caused by moderating variables such as sex, IQ, and personality traits, that moderate the EI–AP association when the same instruments are used (Petrides et al., 2004b; Furnham et al., 2005). Furthermore, they may be due to variations in adaptations to different languages or variations due to cultural differences (Fernández-Berrocal et al., 2005; Ang and van Dyne, 2015). These results go beyond differences between the various instruments to evaluate EI, since they show differences despite using the same instrument. Although it is logical for each theoretical approach to develop and use its own instruments to analyse emotional skills, the results of this type of meta-analysis show the difficulties encountered when comparing the results of studies investigating this area of interest. This is certainly one of the sources of heterogeneity, and the consequent controversy about the results. To clarify this issue, it would be necessary for future studies to select instruments to evaluate emotional skills that have a robust trajectory and well-confirmed psychometric replicative properties in cross-cultural studies. Few studies have been conducted with Spanish-speaking samples. Therefore, more research is needed in Spanish and Latin American population.

The findings of this review should be considered with caution because there were several limitations. The current study was done without controlling for IQ, personality, and other variables that could influence the results. Other studies have been published in languages other than English and Spanish. On the other hand, EI integrates several dimensions, and this study did not take into account the individual associations that each of the dimensions of EI have with AP. It is possible that the associative effect of some dimensions of EI are greater than others, which implies that unifying all the dimensions of EI and analyzing the overall effect they have with AP could produce bias. Future studies should analyze each of the dimensions and their relationship with AP individually, and then compare them to analyze the differences.

These findings have several implications for research and application contexts. The school setting is one of the most important contexts for learning emotional skills and competencies (Zeidner and Matthews, 2016). EI training improves other associated issues, as well as improving performance. Developing emotional skills in early stages of adolescence (Herrera et al., 2020), will allow them to become consolidated personal resources to face risks and promote motivation oriented toward academic success and well-being. For this reason, this review study provides relevant information for the development of programs focused on increasing emotional skills in students, as well as providing tools for teachers and counselors, providing an empirical basis for the development of theoretical educational models oriented to AP. These findings cover the ages at which socio-emotional skills are most important, as well as relevant information for educators and teaching staff on the use of appropriate tools to assess EI in secondary education. We recommend that practitioners be cautious in choosing EI measurement instruments because of differences in their use. In the field of research, this meta-analysis provides information on which future studies should be conducted, helping to clarify the different EI concepts and evaluation measures. Future studies would need to replicate these findings with a larger sample and more of the different EI measures, including variables that may influence AP.

In conclusion, the results of this study found great heterogeneity in the outcomes assessed, so the findings should be considered with caution. The results of this meta-analysis show a moderate association between EI and AP. Future research should explore how other variables influence this relationship, improving our understanding of EI and how it influences our lives. This meta-analytic study presents a quantitative review of the association between EI and AP globally and categorically, shedding light on the gaps in previous studies on the topic on adolescents. This study also shows the inadequacies in the review of studies in this field and provides guidelines to be followed in future empirical studies on AP. These discoveries are of great relevance in the explanatory models intended to predict academic success in secondary education.

## Data Availability Statement

The original contributions presented in the study are included in the article/supplementary material, further inquiries can be directed to the corresponding author/s.

## Author Contributions

All authors listed have made a substantial, direct and intellectual contribution to the work, and approved it for publication.

## Conflict of Interest

The authors declare that the research was conducted in the absence of any commercial or financial relationships that could be construed as a potential conflict of interest.

## Appendix

**Table A1:** Studies included in the meta-analysis of the relationships between EI and AP.

**Study**	**Country**	**Publication type**	**Research design**	**EI measure**	**AP index**	***N***	***Z***	***p***
Abdo (2012)	EEUU	Dissertation	Cross-sectional	EQ-i	GPA	266	0.12	0.025
Abel (2014)	EEUU	Dissertation	Cross-sectional	TEIQue	GPA	78	0.15	0.185
Abdullah et al. (2004)	Malaysia	Article	Cross-sectional	AMEIS	ACS	205	0.14	0.024
Aremu et al. (2006)	Nigeria	Article	Cross-sectional	EIS	AA	500	0.32	0.001
Chamundeswari (2013)	India	Article	Cross-sectional	EIS	GPA	321	0.26	0.010
Clark (2004)	EEUU	Dissertation	Cross-sectional	EQ-i	ABE	161	0.20	0.005
Costa and Faria (2015)	Portugal	Article	Longitudinal	ESCQ	GPA	380	0.17	0.001
Di Fabio and Palazzeschi (2009)^1^	Italy	Article	Cross-sectional	MSCEIT	GPA	124	0.32	0.001
Di Fabio and Palazzeschi (2009)^2^	Italy	Article	Cross-sectional	EQ-i	GPA	124	0.22	0.007
Di Fabio and Palazzeschi (2015)^1^	Italy	Article	Cross-sectional	EQ-i	GPA	133	0.48	0.001
Di Fabio and Palazzeschi (2015)^2^	Italy	Article	Cross-sectional	TEIQue	GPA	133	0.59	0.001
Downey et al. (2008)	Australia	Article	Cross-sectional	SUEIT	GPA	209	0.18	0.031
Downey et al. (2014)	Australia	Article	Cross-sectional	SUEIT	GPA	243	0.26	0.001
Drati (2010)	EEUU	Dissertation	Cross-sectional	EQ-i	AT	165	0.12	0.199
Gil-Olarte Márquez et al. (2006)	Spain	Article	Cross-sectional	MSCEIT	GPA	77	0.50	0.010
Glickman-Rogers (2010)	EEUU	Dissertation	Cross-sectional	MSCEIT	GPA	412	0.33	0.001
Hogan et al. (2010)	Canada	Article	Cross-sectional	EQ-i	GPA	192	0.37	0.008
Jones (2013)	EEUU	Dissertation	Cross-sectional	MSCEIT	GPA	38	0.23	0.082
Jordan et al. (2010)	Ireland	Article	Cross-sectional	EQ-i	AA	86	0.18	0.050
Kaliská (2015)	Eslovaquia	Article	Cross-sectional	TEIQue	MA	169	–0.24	0.001
Khajehpour (2011)	Iran	Article	Cross-sectional	EIS	AA	300	0.45	0.001
Killen (2016)	EEUU	Dissertation	Cross-sectional	EQ-i	GPA	93	0.69	0.001
Kumar et al. (2013)	India	Article	Cross-sectional	EIS	GPA	200	0.27	0.010
Kvapil (2007)	EEUU	Dissertation	Cross-sectional	MSCEIT	GPA	237	0.31	0.001
Lawrence and Deepa (2013)	India	Article	Cross-sectional	TEIQue	AA	400	0.17	0.098
Lui (2009)	India	Dissertation	Cross-sectional	EQ-i	GPA	108	0.03	0.764
MacCann and Fogarty (2011)	EEUU	Article	Cross-sectional	STEM-Y	GPA	293	0.29	0.001
Mateši (2015)	Croatia	Article	Cross-sectional	EQ-i	GA	369	0.01	0.420
Mavroveli et al. (2008)	UK	Article	Cross-sectional	TEIQue	SAT	139	0.07	0.200
Menzie (2005)	EEUU	Dissertation	Cross-sectional	EQ-i	GPA	55	0.21	0.061
Mestre et al. (2006)	Spain	Article	Cross-sectional	MSCEIT	TRA	127	0.33	0.010
Mestre et al. (2006)	Spain	Article	Cross-sectional	SSREI	TRA	127	0.15	0.180
Mitrofan and Cioricaru (2014)	Romania	Conference paper	Cross-sectional	EQ-i	GPA	136	–0.06	0.458
Nelson (2010)	EEUU	Dissertation	Cross-sectional	MSCEIT	VSLECRA	142	0.40	0.001
Ogundokun and Adeyemo (2010)	Nigeria	Article	Cross-sectional	EQBI	WAEC	1563	0.94	0.010
Parker et al. (2004)	Canada	Article	Cross-sectional	EQ-i	GPA	667	0.34	0.010
Petersen (2010)	EEUU	Dissertation	Cross-sectional	EQ-i	AA	51	0.12	0.200
Petrides et al. (2004a)	UK	Article	Cross-sectional	TEIQue	GCSE	650	0.08	0.043
Qualter et al. (2012)	UK	Article	Longitudinal	EQ-i	GCSE	411	0.15	0.080
Rice (2007)	EEUU	Dissertation	Cross-sectional	PSM	GPA	486	0.14	0.001
Vidal Rodeiro et al. (2012)	UK	Article	Cross-sectional	TEIQue	GCSE	874	0.37	0.001
Rodrigo-Ruiz (2017)^1^	Spain	Dissertation	Cross-sectional	STEM-Y	GPA	232	0.22	0.001
Rodrigo-Ruiz (2017)^2^	Spain	Dissertation	Cross-sectional	MSCEIT	GPA	232	0.35	0.001
Rodrigo-Ruiz (2017)^3^	Spain	Dissertation	Cross-sectional	TEIQue	GPA	232	0.29	0.001
Trigueros et al. (2019)	Spain	Article	Cross-sectional	EIS	GPA	615	0.69	0.001
Usán Supervía and Salavera Bordás (2018)	Spain	Article	Cross-sectional	TMMS	GPA	3512	0.22	0.001
Usán and Salavera (2019)	Spain	Article	Cross-sectional	TMMS	GPA	1756	0.12	0.001
Woitaszewski and Aalsma (2004)	EEUU	Article	Cross-sectional	MEIS	GPA	39	0.50	0.090
Xu (2018)	China	Article	Cross-sectional	EM	GA	1799	–0.03	0.101

1 First instrument used,

2 second instrument used,

3 *third instrument useds*.
